# Microwave Tunneling and Robust Information Transfer Based on Parity-Time-Symmetric Absorber-Emitter Pairs

**DOI:** 10.34133/2019/7108494

**Published:** 2019-11-28

**Authors:** Zhicheng Xiao, Younes Ra'di, Sergei Tretyakov, Andrea Alù

**Affiliations:** ^1^Department of Electrical and Computer Engineering, The University of Texas at Austin, Austin, TX 78712, USA; ^2^Advanced Science Research Center, City University of New York, New York, NY 10031, USA; ^3^Department of Electronics and Nanoengineering, Aalto University, FI-00076 Aalto, Finland

## Abstract

Robust signal transfer in the form of electromagnetic waves is of fundamental importance in modern technology, yet its operation is often challenged by unwanted modifications of the channel connecting transmitter and receiver. Parity-time- (PT-) symmetric systems, combining active and passive elements in a balanced form, provide an interesting route in this context. Here, we demonstrate a PT-symmetric microwave system operating in the extreme case in which the channel is shorted through a small reactance, which acts as a nearly impenetrable obstacle, and it is therefore expected to induce large reflections and poor transmission. After placing a gain element behind the obstacle, and a balanced lossy element in front of it, we observe full restoration of information and overall transparency to an external observer, despite the presence of the obstacle. Our theory, simulations, and experiments unambiguously demonstrate stable and robust wave tunneling and information transfer supported by PT symmetry, opening opportunities for efficient communication through channels with dynamic changes, active filtering, and active metamaterial technology.

## 1. Introduction

Information transfer in the form of electromagnetic waves is ubiquitous in today's world, from free space (wireless) to transmission line (guided) channels [1–5]. The channels connecting a transmitter and a receiver are typically time-varying and are affected by the presence of various obstacles. Therefore, the wave must tunnel through nonideal channels, with strong insertion loss. Resonant transmission, such as electron tunneling through quantum wells, resonant photon tunneling through optical barriers, and microwave tunneling through extremely squeezed or bent channels can address this issue to some extent, but typically with severe trade-offs in terms of bandwidth, sensitivity, and complexity, among others [6–9]. In most practical scenarios, dynamic and complex electromagnetic environments pose challenges to the system operation and make these solutions even more challenging. For instance, in applications where dynamic obstacles are inevitable, like communicating with biomedical implants such as artificial cardiac pacemakers [10] or interrogating embedded health sensors in civil infrastructures [11], engineers need to adaptively tune complex matching networks or the operational frequency to maximize the transmission efficiency. In extreme scenarios, the channel between a transmitter and a receiver may be completely blocked, which cannot be addressed by dynamically tuning the matching network. In such practical scenarios, transferring the signal over the channel may be impossible.

In a recent theoretical work [12], we showed that in such systems instead of transferring the input signal to the receiving port through a channel with dynamic changes, one can just transfer little information about the input signal during the transient and replicate the entire signal at the output port using this information based on a different form of signal tunneling driven by parity-time (PT) symmetry. Quite interestingly, in contrast to conventional solutions, in such a system, the input and output ports do not have to stay connected throughout the whole information transfer process.

Here, we experimentally prove that, by placing a shunt lossy element in the transmitting node before the obstacle and a judiciously designed gain element in the receiving node, forming a PT-symmetric conjugate pair [13–31], we can restore full transmission at the operational frequency, despite the presence of a nearly unitary reflective obstacle blocking the transmission channel. The obstacle in our experiment is modeled as a shorting element along a transmission line. The lossy element paired with the obstacle is designed to behave as a Salisbury screen absorber [32], while the gain element acts as a synchronized emitter operating as the time-reversed version of a Salisbury absorber. This PT-symmetric wave tunneling and information transfer system enables robust full-wave transmission through an otherwise impenetrable barrier.

## 2. Results

### 2.1. PT-Symmetric Wave Tunneling and Information Transfer

**Figure 1:**
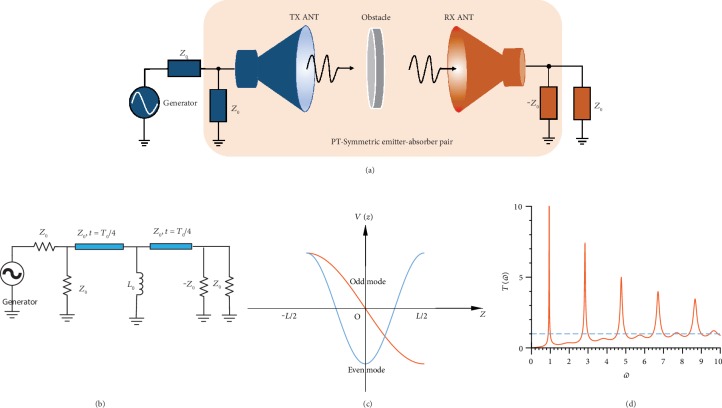
PT-symmetric wave tunneling model, mode profile, and power transmission characteristics. (a) PT-symmetric wave tunneling and information transfer scheme. A highly reflective obstacle blocks the channel. A gain element −*Z*_0_ is placed behind this obstacle, and a lossy element *Z*_0_ is placed before the obstacle to facilitate full-wave transmission. (b) A simplified wired version of the PT-symmetric robust information transfer scheme based on a transmission line. The obstacle is modeled as a small inductive element *L*_0_. (c) Fundamental odd and even mode profiles of the PT-symmetric absorber-emitter pair. They are significantly different from the Hermitian system due to the Neumann boundary condition and PT symmetry. The points *Z* = −(*L*/2) and *Z* = *L*/2 mark the location of the loss and gain unit, where *L* is the electrical length of the transmission connecting these two elements. (d) Tunneling characteristics of the PT-symmetric system. The *π* phase transmission maxima locate at *ω* = (2*k* + 1)*ω*_0_,  *k* = 0, 1, 2, 3⋯, and the in-phase transmission peaks are determined by the transcendental equation (1/2*α*)(*ω*/*ω*_0_)^−1^ = tan(*πω*/2*ω*_0_), where *α* = *ω*_0_*L*_0_/*Z*_0_ is the coupling strength between the absorber and emitter and *ω*_0_ = 2*π*/*T*_0_ is the fundamental resonant frequency.

PT-symmetric tunneling and robust information transfer are schematically sketched in Figure 1(a), where the signal is transported wirelessly via an air channel from a transmitting to a receiving antenna. When a reflective obstacle is inserted within the transmission channel, information transfer efficiency is expected to drop dramatically. To restore full-wave transmission, in this work, we add a gain element −*Z*_0_ behind the reflecting obstacle and a conjugate lossy element *Z*_0_ in front of it to form a PT-symmetric conjugate pair, where *Z*_0_ is the characteristic impedance of the transmission channel (see Figure 1(a)). As we show in the following, this combination can largely restore transmission through the channel, despite the presence of the largely reflective obstacle. In order to prove this principle, we model the wireless information transmission channel with a basic transmission line, as shown in Figure 1(b), and the highly reflective obstacle with a small inductor *L*_0_, close to a short and therefore highly reflective. Since we utilize the resonant tunneling feature of the PT-symmetric system, the transmission line is set at half-wavelength of the impinging wave. As a result, the loss element *Z*_0_, the first quarter-wavelength transmission channel, and the highly reflective obstacle behave like a Salisbury screen absorber. The obstacle and the second quarter-wavelength transmission channel, together with the gain element −*Z*_0_, work as a synchronized information transmitter, a time-reversed replica of a Salisbury absorber, which emits in sync with the absorbing portion. We show that in a steady state the whole PT-symmetric system works as a tunneling channel for impinging waves, mimicking a lossless, ideally transmitting channel.

To begin with, we analyze the resonant transmission mode and frequency of the PT-symmetric wave tunneling and information transfer system. We define the steady-state voltage on the transmitting node, obstacle, and receiving node as *V*_source_, *V*_obstacle_, and *V*_load_, which form the state vector |*ψ*〉 = [*V*_source_ *V*_obstacle_ *V*_load_]^T^. The characteristic equation reads PT|*ψ*〉 = *λ*|*ψ*〉, which supports even and odd eigenstates $|\psi_{+}\rangle ={\left[\begin{array}{ccc} 1 & -1 & 1 \end{array}\right]}^{\text{T}}$ and $|\psi_{-}\rangle ={\left[\begin{array}{ccc} 1 & 0 & -1 \end{array}\right]}^{\text{T}}$ and eigenvalues *λ*_±_ = ±1, where

(1) $$\begin{array}{c} P=\left[\begin{array}{ccc} 0 & 0 & 1\\ 0 & 1 & 0\\ 1 & 0 & 0 \end{array}\right] \end{array}$$

is the parity operator, *T* is the time reversal operator (equivalent to complex conjugation for a steady state), and T is the transpose operator. As we can infer from the above analysis, the obstacle is a short and contributes no phase delay at odd resonant tunneling states. Therefore, the total phase delay of the transmitted wave is *ω*(*T*_0_/2) = (2*k* + 1)*π* with *ω* = (2*k* + 1)*ω*_0_, *k* = 0, 1, 2, 3⋯, and *ω*_0_ = 2*π*/*T*_0_ being the resonant frequency of the fundamental standing mode. For the even scattering state, we sum up the phase delay on the transmission line and the inductor:

(2) $$\begin{array}{c} -\omega \frac{T_{0}}{4}+2\arctan\frac{Z_{0}}{2\omega L_{0}}-\omega \frac{T_{0}}{4}=2k\pi , \end{array}$$

which can be simplified to the characteristic equation

(3) $$\begin{array}{c} \frac{1}{2\alpha }{\left(\frac{\omega }{\omega_{0}}\right)}^{-1}=\tan\left(\frac{\pi }{2}\frac{\omega }{\omega_{0}}\right), \end{array}$$

where *α* = *ω*_0_*L*_0_/*Z*_0_ is the coupling strength between the Salisbury screen absorber and its conjugate emitter and *Z*_0_ is the characteristic impedance of the transmission line. The eigenequation of even standing modes is a transcendental equation, which requires a numerical evaluation. As *α* approaches zero, the circuit is shorted by the obstacle and the wave cannot tunnel through the system; as *α* approaches infinity, the load can be neglected and the even mode has the close-form solution *ω* = 2*kω*_0_, *k* = 0, 1, 2, 3⋯. An alternative approach to find the resonant frequencies of the system is to employ the transfer matrix method and the generalized conservation law, with detailed calculations presented in the Supplementary Materials. The standing even/odd mode solutions |*ψ*_±_〉 explicitly show that the absorber and emitter exhibit identical amplitudes and 0/*π* phase difference, which is direct evidence of synchronized wave absorption at the input port and emission at the output port. We demonstrate the standing wave profile of this PT-symmetric absorber-emitter pair in Figure 1(c). The unusual standing mode profile, considering the presence of the reflective obstacle, is a direct manifestation of PT symmetry and Neumann boundary conditions at the ports. We also show the power transmission spectrum in Figure 1(d), which demonstrates full-wave tunneling at the resonant frequencies. The tunneling mechanism satisfies the generalized flux conservation law applicable to any two-port PT-symmetric network [33], which reads *T* − 1 = |*r*_L_*r*_R_|*e*^*i*(*ϕ*_L_ − *ϕ*_R_ + *π*)^, where *T* is the power transmission coefficient, *r*_L_ and *r*_R_ are the amplitude reflection coefficients on the left and right ports of the system, and *ϕ*_L_ and *ϕ*_R_ are the corresponding phases of the amplitude reflection coefficients. As a result, the system demonstrates superunitary power transmission inside the region bounded by any pair of even and odd tunneling eigenfrequencies. Note that, due to the presence of gain in the system, the transmission may go over unity at frequencies close to the tunneling frequency; however, the system remains stable.

To better understand the PT-symmetric wave tunneling, it is of interest to investigate the scattering properties at resonance and analyze the PT phase transition of the scattering state. Rigorous transfer matrix analysis enables us to express the scattering properties of even and odd resonant states of this two-port information transfer network as

(4) $$\begin{array}{c} S_{\pm }=\left[\begin{array}{cc} 0 & \pm 1\\ \pm 1 & \pm \frac{2j}{\alpha }{\left(\frac{\omega }{\omega_{0}}\right)}^{-1} \end{array}\right], \end{array}$$

where *S*_+_ and *S*_−_ are scattering matrices of even and odd resonant modes, respectively, obeying the symmetry relation *PS*_±_^∗^(*ω*)*PS*_±_(*ω*) = **I** [29]. Wave tunneling happens at the resonant frequencies, and it is totally independent of the obstacle strength *α* or its type (inductive, capacitive, even resistive, or any combination of these) between the Salisbury absorber and the synchronized emitter, since the obstacle is effectively shorted in a steady state. This offers unique flexibility to maintain the transmissivity of the proposed system for arbitrary variations of the obstacle, ensuring stable operation. Even for dynamically varying obstacles, the system is capable of self-tuning to a stable full-transmission region (see Supplementary Materials).

The scattering matrix in the equation has two eigenvalues $\lambda_{1,2}=\left(-j\pm \sqrt{{\left(\alpha \left(\omega /\omega_{0}\right)\right)}^{2}-1}\right)/\left(\alpha \left(\omega /\omega_{0}\right)\right)$. At *α* = (*ω*/*ω*_0_)^−1^, these two eigenvalues coalesce, supporting a non-Hermitian degeneracy. This exceptional point (EP) separates the scattering system from its PT-symmetric phase when *α* > (*ω*/*ω*_0_)^−1^ and its broken PT-symmetric phase when *α* > (*ω*/*ω*_0_)^−1^. We substitute the eigenfrequencies of even and odd scattering states *α* < (*ω*/*ω*_0_)^−1^ into the expression *α* = (*ω*/*ω*_0_)^−1^ to find the EPs:

(5) $$\begin{array}{c} \alpha_{\text{EP}}^{\text{Even}}=\frac{\pi }{2\arctan1/2+2k\pi },\;k=0,1,2\cdots ,\\ \alpha_{\text{EP}}^{\text{Odd}}=\frac{1}{2k+1},\;k=0,1,2\cdots . \end{array}$$

Depending on the reflectivity of the obstacle, i.e., how close to zero is its reactance, the resonant tunneling state can be in the exact PT symmetry phase with unitary eigenvalues |*λ*_1_| = |*λ*_2_| = 1 or in the broken PT symmetry phase with |*λ*_1_| = 1/|*λ*_2_| > 1 [29]. The PT tunneling functionality is independent of the PT symmetry phase transition point. The stability analysis is presented in the Supplementary Materials, which shows inherent stability for any finite value of the obstacle reflectivity [0 < *α*<∞].

Quite interestingly, the proposed system is robust even when the time delay on the transmission line is asymmetric (see the detailed discussion in the Supplementary Materials). As it was mentioned above, regardless of how small or large the inductive load is (i.e., how reflective the inductive load is), the structure will still fully retrieve the input signal at the output once it reaches a steady state. In fact, the obstacle can be inductive, capacitive, or even resistive, and yet the PT-symmetric setup will still recover the input signal at the output. In the general case of a finite-size obstacle, the 1-D transmission line model implemented here does not apply, because of scattering into the continuum of radiation. However, if the beamwidth of the input wave is smaller than the size of the obstacle and the obstacle does not scatter energy from its edges, scattering loss can be modeled as a small resistive load in our transmission line model and we still expect to efficiently recover the input signal at its output.

### 2.2. Experimental Realization and Robustness Analysis

**Figure 2:**
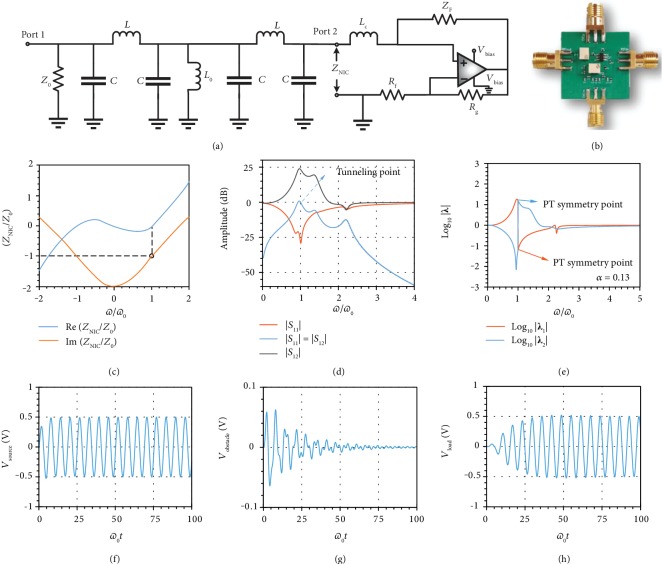
Realistic design and implementation of the PT-symmetric wave tunneling prototype. (a) Circuit schematic. Transmission line is replaced with a *π*-type transmission line which consists of inductor *L* and capacitor *C*. (b) Photograph of the fabricated PCB prototype. Two big white components are tunable resistors. The black component with six pins is the OPA 355-Q1 amplifier. Left and right ports are source port 1 and load port 2 in the schematic. Upper and lower ports are DC bias ports for the amplifier. (c) Dispersion of the impedance of the gain element. Black circle marks the operational point. (d) ADS and Modelithics simulation of the amplitude of scattering parameters. Tunneling point is marked in the figure. (e) Spectral properties of eigenvalues of the scattering matrix. Exact PT symmetry is achieved at tunneling frequency where eigenvalues obey unitary condition |*λ*_1_(*ω*_0_)*λ*_2_(*ω*_0_)| = 1. The coupling coefficient *α* is 0.13, ensuring robust operation of the whole circuit in the presence of the obstacle. (f) Numerical transient response at the source port where full absorption is achieved at tunneling frequency. The generator voltage is 1 volt. (g) Numerical transient response at the obstacle which is short in the steady state. (h) Numerical transient response at the load port where full-wave tunneling is observed in the steady state.

We experimentally demonstrate a robust proof-of-concept PT-symmetric microwave tunneling prototype (see Figures 2(a) and 2(b)). The gain unit is realized through a dispersive negative impedance converter (NIC) based on a noninverting feedback amplifier configuration (see Figure 2(a)). A compensating inductor *L*_c_ in series with the noninverting port realizes purely negative impedance −*Z*_0_ at the operational frequency. The dispersion relation of the NIC follows

(6) $$\begin{array}{c} Z_{\text{NIC}}\left(s\right)=\frac{s+A_{0}{\beta \omega }_{\text{p}}}{s+A_{0}\left(\beta -1\right)\omega_{\text{p}}}Z_{\text{F}}+{sL}_{\text{c}}, \end{array}$$

where *A*_0_ is the open-loop gain of the amplifier, *ω*_p_ is the pole frequency of the amplifier, *β* = *R*_f_/(*R*_f_ + *R*_g_) is the feedback factor, *Z*_F_ = 2*Z*_0_ is the feedback resistor, and *s* is the complex frequency. We choose a compensating inductor *L*_c_ = 3*Z*_0_/*ω*_3dB_ and the operational frequency $\omega_{0}=\omega_{3\text{dB}}/\sqrt{3}$, where *ω*_3dB_ = *A*_0_*ω*_p_/2 is the 3 dB frequency of the closed-loop gain coefficient. The NIC dispersion is shown in Figure 2(c), showing a negative impedance −*Z*_0_ at the operational frequency *ω*_0_.

Our prototype is designed to work at the fundamental odd scattering state. We realize the transmission line through a *π*-type LC resonator to reduce the form factor (see Figure 2(a)), which offers unitary transmission as well as *π*/2 phase delay at resonant frequency to mimic a quarter-wavelength transmission line segment. The impedance is automatically matched at both transmitting and receiving nodes, as we choose *L* = *Z*_0_/*ω*_0_ and *C* = 1/*Z*_0_/*ω*_0_. This compact design significantly reduces the form factor of our device from *λ*/2 to a deeply subwavelength scale *λ*/50. The fabricated device is shown in Figure 2(b), with a size of approximately 2 cm by 2 cm.

We confirm our theoretical analysis with a cosimulation between ADS and the Modelithics package, which shows excellent PT-symmetric tunneling at 48.7 MHz. The scattering properties are demonstrated in Figure 2(d) with a tunneling point of -0.01 dB transmission and -28.8 dB reflection. Finally, we confirm the PT symmetry of our designed circuit by graphing the spectral properties of the eigenvalues of the scattering matrix in Figure 2(e). With the coupling coefficient *α* = 0.13, the eigenvalues demonstrate unitary property |*λ*_1_| = 1/|*λ*_2_| at *ω*_0_, which is a typical hallmark of PT symmetry in the broken symmetry phase. No other PT symmetry point is found in the full spectra.

To better understand how this PT-symmetric wave tunneling circuit works, we plot the temporal response at the source, obstacle, and load nodes in Figures 2(f)–2(h). We place a microwave generator *V*_g_ = sin*ω*_0_*t* with internal impedance *Z*_0_ in the source node. The voltage at this port initially experiences a small reflection and then rapidly reaches a steady state *V*_source_ = (1/2)sin*ω*_0_*t*, indicating that the impinging wave is fully absorbed by the shunt resistor *Z*_0_. The obstacle node demonstrates a purely decaying response, confirming our previous steady-state analysis in the ideal PT-symmetric configuration *V*_obstacle_ = 0. The load port is essentially an emitter, and it demonstrates an exponentially growing trend towards a steady state *V*_load_ = −(1/2)sin*ω*_0_*t*. Since it operates at the fundamental odd resonant tunneling state, the emitted wave has a *π* phase shift with respect to the impinging wave.

**Figure 3:**
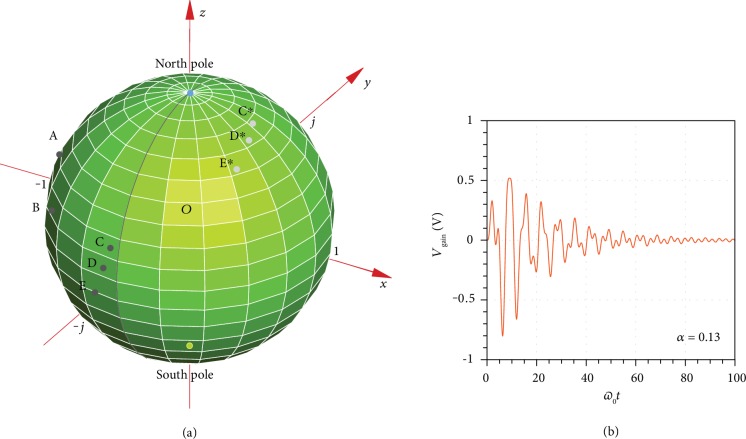
Pole diagram and impulse response. (a) Pole distribution of the transfer function on a Riemann surface. Grey solid line (prime meridian) marks the watershed between stable and unstable regions. The north pole is the pole in the case *α* = ∞, corresponding to an open obstacle and leading to marginally stable operation. For the prototype, we fabricated *α* = 0.13; there are eight poles in the transfer function, A, B, C, D, E, C^∗^, D^∗^, and E^∗^, where C^∗^, D^∗^, and E^∗^ are the conjugates of C, D, and E, respectively. A and B are located on the 90-degree west longitude; C, D, and E are located close to the prime meridian but to the left; C^∗^, D^∗^, and E^∗^ are located in the back surface. All these eight poles are in the stable region. (b) Numerical impulse response at the gain unit. The input port is excited with a pulse *V*_generator_ = *δ*(*t*).

Apart from the PT-symmetric full-wave tunneling functionality, our design is stable and robust to parameter detuning and reasonable fabrication errors [34–36]. More interestingly, our system can self-tune to the stable region even if the obstacle dynamically changes in time (see Supplementary Materials). To study the stability issue, it is important to investigate the voltages at the source, obstacle, and load in the complex frequency domain. Application of Kirchhoff's current and voltage laws leads to a set of linear equations:

(7) $$\begin{array}{c} \left[\begin{array}{ccc} 2+\frac{s}{\omega_{0}}+{\left(\frac{s}{\omega_{0}}\right)}^{-1} & -{\left(\frac{s}{\omega_{0}}\right)}^{-1} & 0\\ {\left(\frac{s}{\omega_{0}}\right)}^{-1} & -2\frac{s}{\omega_{0}}-\left(1+\frac{1}{\alpha }\right){\left(\frac{s}{\omega_{0}}\right)}^{-1} & {\left(\frac{s}{\omega_{0}}\right)}^{-1}\\ 0 & {\left(\frac{s}{\omega_{0}}\right)}^{-1} & -\frac{s}{\omega_{0}}-{\left(\frac{s}{\omega_{0}}\right)}^{-1}-\frac{Z_{0}}{Z_{\text{NIC}}} \end{array}\right]\left[\begin{array}{c} {\tilde{V}}_{\text{source}}\left(s\right)\\ {\tilde{V}}_{\text{obstacle}}\left(s\right)\\ {\tilde{V}}_{\text{load}}\left(s\right) \end{array}\right]=\left[\begin{array}{c} {\tilde{V}}_{\text{g}}\left(s\right)\\ 0\\ 0 \end{array}\right], \end{array}$$

where ${\tilde{V}}_{\text{g}}\left(s\right)$ is the voltage of the microwave generator and ${\tilde{V}}_{\text{source}}\left(s\right)$, ${\tilde{V}}_{\text{obstacle}}\left(s\right)$, and ${\tilde{V}}_{\text{load}}\left(s\right)$ are the complex voltages on the source, obstacle, and load nodes, respectively. Here, we define the transfer function as the voltage ratio between the load and generator: $\tilde{H}\left(s\right)={\tilde{V}}_{\text{load}}\left(s\right)/{\tilde{V}}_{\text{g}}\left(s\right)$ (see the detailed expression in Materials and Methods). To maintain a stable operation, the impulse response should have a finite energy, which is equivalent to requiring that all the poles of the transfer function lie on the left hemi-Riemann sphere [36]. Equation (7) indicates that the relative pole locations only depend on the strength of the obstacle. As *α* approaches to zero, the transfer function turns to zero and it is meaningless to study the stability issue. As *α* goes to infinity, the emitter is directly connected with the absorber, creating a marginally stable point on the north pole (see Figure 3(a)). In this scenario, any small perturbation in the system will move the marginally stable point and transform it into eight poles on the right hemi-Riemann sphere. Numerical computation indicates that our circuit operates robustly as the coupling strength varies between 0.1 and 0.3. As our realistic design involves both dispersive negative feedback and a *π*-type transmission line, they place a more stringent condition on stability compared with the ideal model where the stable condition is 0 < *α*<∞. In our fabricated prototype, we carefully choose *α* = 0.13 to ensure a stable operation within expected fabrication imperfections. Figure 3 demonstrates the pole distributions as well as the impulse response of our design. There are eight poles on the left hemi-Riemann sphere when *α* = 0.13, indicating that our fabricated prototype is stable. To further confirm the robustness of our implemented prototype, we study the influence of feedback factor *β* and feedback resistor *Z*_F_ perturbation on the pole distribution and impulse responses in Materials and Methods.

### 2.3. Observation of PT Tunneling and Information Transfer

**Figure 4:**
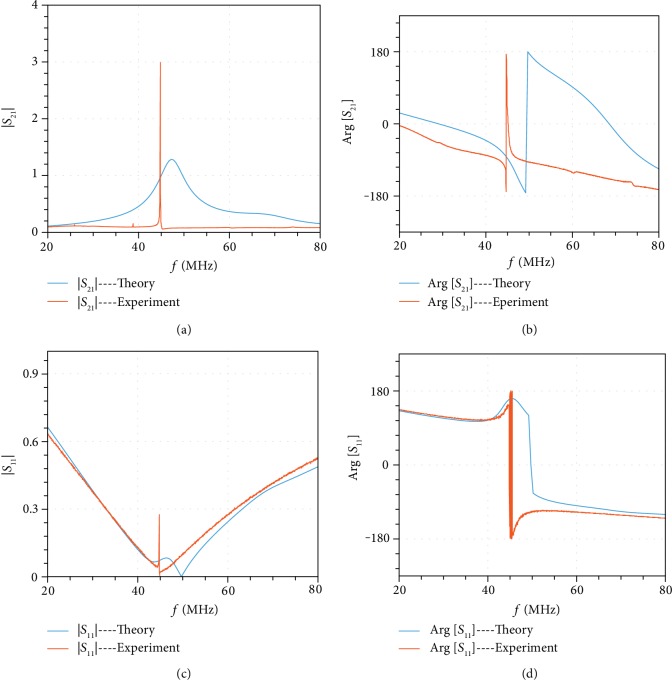
Measurements of the amplitude transfer characteristics and reflection coefficient. (a) Forward transmission spectrum. Resonant tunneling occurs at 44.8 MHz. (b) Phase diagram of the transmission coefficient. Tunneling field experiences *π* phase shift at resonant frequency. (c) Reflection at the input port. -50 dB reflection is observed at 44.8 MHz, indicating that the matching network works quite well. (d) Phase diagram of reflection coefficient at the input port. Measured data in a wider (20-200 MHz) and narrower (40-50 MHz) spectrum are presented in the extended data Figs. [Supplementary-material supplementary-material-1] and [Supplementary-material supplementary-material-1] in the supplementary document.

Figures 4(a) and 4(b) show the experimentally measured and theoretically calculated forward transmission spectrum and phase. A PT-symmetric unitary transmission was observed at 44.8 MHz with a 180-degree phase, which agrees with our simulation. Meanwhile, the reflection is fully suppressed at this point, as shown in Figures 4(c) and 4(d). The experimental reflection phase at resonance is essentially undefined due to the zero amplitude, causing fast oscillations around the resonance, as shown in Figure 4(d). It is worth mentioning that at resonance, the experimental backward reflection amplitude |*S*_22_| is 15 dB and the phase Arg[*S*_22_] is -90 degrees, confirming that our PT-symmetric system has asymmetric reflections and satisfies the generalized conservation law. Since the system is linear and time-invariant, reciprocity is satisfied and the transmission coefficients in forward and backward directions are identical. The observed tunneling frequency is smaller than the simulation results, which may stem from fabrication errors where the inductance or capacitance values in the transmission line are smaller than the designed ones. The scattering parameters in a narrower or wider spectrum are demonstrated in the extended data figure section in the Supplementary Materials (Figs. [Supplementary-material supplementary-material-1]). In the extended Fig. [Supplementary-material supplementary-material-1], we show the linearity of our tunneling circuit, which demonstrates an excellent linear relation between input and output power at tunneling frequency. This graph clearly shows that although at the steady state, the output is completely disconnected from the input (since the inductive load is fully shorted), the output can still linearly follow the input. In other words, if after reaching the steady state, any change happens to the input signal, the system goes into the transient regime for a very short period of time, during which the information of the input signal is communicated towards the output port and then the output reaches the steady state again and fully replicates the input signal.

## 3. Discussion

In conclusion, we have demonstrated microwave tunneling and information transfer through a PT-symmetric absorber-emitter pair. Our study represents a landmark towards realistic implementation of information transfer systems with extreme robustness, able to tunnel the input signal through otherwise impenetrable obstacles with large robustness, and it shows promises to spawn a series of applications. For instance, our loss-neutral-gain arrangement exhibits a third-order exceptional point in the bound state. By properly designing the system at this higher-order exceptional point, the eigenfrequency splitting of the corresponding Hamiltonian matrix shows enhanced high sensitivity proportional to the cubic root of the perturbation strength on the system, which is very favorable to design ultrasensitive microsensors. Meanwhile, this prototype can be used as a bandpass active filter which allows for simultaneous narrowband signal filtering and amplification. Furthermore, our design provides relevant insights into realization of active cloaking devices and active metasurfaces which exhibit unique properties not available in passive counterparts. In summary, the design strategies and stability analysis in this work pave the way towards future realizations of PT-symmetric functionalities in optics and microwave regimes. Considering the extensive connections between electromagnetic, mechanic, and matter waves, our study can also spur practical applications of PT symmetry in these other fields of research.

## 4. Materials and Methods

### 4.1. Circuit Design and Fabrication

The design of the circuit and fabricated prototype is shown in Figures 2(a) and 2(b). In the NIC part, we use the amplifier OPA 355-Q1 from Texas Instruments. This amplifier has a 200 MHz gain bandwidth product, with an open-loop gain *A*_0_ = 10^5^ and pole frequency *ω*_p_ = 2*π* × 2000 Hz. The effective impedance of the NIC in the Fourier domain can be easily inferred from Equation (6) by replacing complex frequency *s* with physical frequency *jω*:

(8) $$\begin{array}{c} Z_{\text{NIC}}=Z_{\text{F}}\frac{\omega^{2}+{\left(A_{0}\omega_{\text{p}}\right)}^{2}\beta \left(\beta -1\right)}{\omega^{2}+{\left[A_{0}\left(\beta -1\right)\omega_{\text{p}}\right]}^{2}}+j\left\{\frac{-\omega A_{0}\omega_{\text{p}}Z_{\text{F}}}{\omega^{2}+{\left[A_{0}\left(\beta -1\right)\omega_{\text{p}}\right]}^{2}}+\omega L_{\text{c}}\right\}. \end{array}$$

To have purely negative impedance −*Z*_0_, the real part of the above equation should be −*Z*_0_ and the imaginary part of the effective impedance should be 0. Then, we have the following operational conditions:

(9) $$\begin{array}{c} \omega =A_{0}\omega_{\text{p}}\sqrt{\frac{\left(1-\beta \right)\left[Z_{\text{F}}\beta -Z_{0}\left(1-\beta \right)\right]}{Z_{\text{F}}+Z_{0}}}, \end{array}$$

(10) $$\begin{array}{c} L_{\text{c}}=\frac{Z_{\text{F}}+Z_{0}}{\left(1-\beta \right)A_{0}\omega_{\text{p}}}, \end{array}$$

where *Z*_0_ = 50 *Ω*, *Z*_F_ = 100 *Ω*, *β* = *R*_f_/(*R*_f_ + *R*_g_) = 0.5, and *R*_f_ = *R*_g_ = 560 *Ω*. Here, the feedback resistor *Z*_F_ is chosen as 2*Z*_0_ due to the dispersion of the one-pole model amplifier. We substitute these values into Equation (9) and get the operational frequency *f* = *ω*/2*π* = 57.74 MHz of the negative impedance converter and the compensating inductor *L*_c_ = 238.7 nH. In circuit simulation, we use two inductors with inductance value 120 nH. Then, we cosimulate our circuit with ADS and Modelithics to check the effective negative impedance. Our simulations demonstrate a −50.9 *Ω* negative impedance at 50.2 MHz. The shift of the design frequency can be contributed to the parasitic effect and possible high-order poles in the amplifier. The inductor *L* = 150 nH and capacitor *C* = 68 pF in the transmission line are chosen to match both the operational frequency of NIC and the characteristic impedance of the port. The reflective obstacle is modeled as an inductor *L*_0_ = 20 nH, leading to a coupling factor *α* = 0.13 and ensuring robust operation of the system.

The circuit is fabricated on a 0.062-inch-thick FR-4 substrate with relative permittivity 4.4 and dissipation factor 0.017. The following components are surface mounted on the PCB board: (1) characteristic impedance *Z*_0_ from KOA Speer with part number RK73B1ETTP510J; (2) feedback resistor *Z*_F_ from KOA Speer with part number RK73B1ETTP910J; (3) feedback resistor *R*_f_ from KOS Speer with part number RK73B1ETTP561J; (4) two variable resistors from Bourns Inc. with part numbers 3223W-1-101ETR-ND and 3223W-1-200ETR-ND; (5) capacitor in the resonator from Murata Inc. with part number GRM1552C2A680GA01#; (6) inductors from Coilcraft with part numbers 0402HPHR15X, 0402CS20N, and 0402CS12X; (7) amplifier from Texas Instruments with part number OPA355QDBVRQ1; and (8) port connectors from Amphenol with part number 132322.

### 4.2. Stability Analysis

A linear, time-invariant, causal circuit is stable if and only if the impulse response is absolutely integrable, which means

(11) $$\begin{array}{c} \int_{0}^{\infty }\left|H\left(t\right)\right|dt=\text{finite}, \end{array}$$

where *H*(*t*) = ∑_*n*=0_^∞^*c*_*n*_*e*^*p*_*n*_*t*^ is the impulse response, *p*_*n*_ is the nth pole of the transfer function *H*(*s*) = ∑_*n*=0_^∞^*c*_*n*_/(*s* − *p*_*n*_), *c*_*n*_ is the amplitude coefficient of each Laplace component [36]. Therefore, to operate in the stable region, all the real parts of the poles must be negative:

(12) ∀n,Repn<0,

which is the stability condition of a linear, causal, and time-invariant circuit.

In our circuit, we assume that the system is excited with a power source *V*_g_ which has an internal impedance *Z*_0_. The transfer function $\tilde{H}\left(s\right)$ can be defined as the ratio between ${\tilde{V}}_{\text{load}}\left(s\right)$ and ${\tilde{V}}_{\text{g}}\left(s\right)$. According to Kirchhoff's current and voltage laws, we express currents and voltages in the Laplace domain:

(13) $$\begin{array}{c} I_{1}={\tilde{V}}_{\text{source}}\left(\frac{1}{Z_{0}}+sC\right)+I_{2},\\ I_{2}={\tilde{V}}_{\text{obstacle}}\left(2sC+\frac{1}{{sL}_{0}}\right)+I_{3},\\ I_{3}={\tilde{V}}_{\text{load}}\left(sC+\frac{1}{Z_{0}}+\frac{1}{Z_{\text{NIC}}}\right),\\ \;{\tilde{V}}_{\text{g}}-{\tilde{V}}_{\text{source}}=I_{1}Z_{0},\;{\tilde{V}}_{\text{source}}-{\tilde{V}}_{\text{obstacle}}=I_{2}sL,\;{\tilde{V}}_{\text{obstacle}}-{\tilde{V}}_{\text{load}}=I_{3}sL, \end{array}$$

where $Z_{\text{NIC}}=2Z_{0}\left[\left(s+\sqrt{3}\omega_{0}\right)/\left(s-\sqrt{3}\omega_{0}\right)+\sqrt{3}s/2\omega_{0}\right]$. We solve the above linear equation set and get the transfer function:

(14) $$\begin{array}{c} \tilde{H}\left(s\right)=\frac{s}{\omega_{0}}\alpha \left(2\sqrt{3}-\frac{s}{\omega_{0}}+\sqrt{3}{\left(\frac{s}{\omega_{0}}\right)}^{2}\right){\left\{2\sqrt{3}+\left[5\sqrt{3}\left(\alpha +1\right)-1\right]\frac{s}{\omega_{0}}+\left(7\sqrt{3}-2-2\alpha \right){\left(\frac{s}{\omega_{0}}\right)}^{2}+\left(8\sqrt{3}-2-4\alpha +23\sqrt{3}\alpha \right){\left(\frac{s}{\omega_{0}}\right)}^{3}+\left(6\sqrt{3}-2-8\alpha +24\sqrt{3}\alpha \right){\left(\frac{s}{\omega_{0}}\right)}^{4}+\left(3\sqrt{3}-1-6\alpha +22\sqrt{3}\alpha \right){\left(\frac{s}{\omega_{0}}\right)}^{5}+\left(\sqrt{3}-4\alpha +14\sqrt{3}\alpha \right){\left(\frac{s}{\omega_{0}}\right)}^{6}+\left(6\sqrt{3}\alpha -2\alpha \right){\left(\frac{s}{\omega_{0}}\right)}^{7}+2\sqrt{3}\alpha {\left(\frac{s}{\omega_{0}}\right)}^{8}\right\}}^{-1}. \end{array}$$

Simple numerical calculation is employed to solve the pole locations of the above transfer function. Our calculation indicates that for stable operation of the PT-symmetric circuit, the coupling coefficient must meet the following condition:

(15) $$\begin{array}{c} 0.1<\alpha <0.3. \end{array}$$

To verify the robustness of our PT-symmetric wave tunneling circuit, we investigate the influence of parameter detuning on pole locations. Extended data Fig. [Supplementary-material supplementary-material-1] demonstrates pole locations with feedback factor detuning and feedback resistance detuning, which shows excellent stability under reasonable perturbation.

It is also equally important to investigate the impulse response and confirm the stability of our PT-symmetric system in the time domain. We substitute the solutions of the poles into the following equation of impulse response:

(16) $$\begin{array}{c} H\left(t\right)=\overset{\infty }{\underset{n=1}{\sum }}\text{R}\text{es}{\left[e^{\text{st}}H\left(s\right)\right]}_{n}, \end{array}$$

where “Res” means the residue of a complex function. Extended data Fig. [Supplementary-material supplementary-material-1] in the supplementary document demonstrates the impulse responses with parameter detuning, which shows that the impulse response has a finite energy and evolves in a stable fashion in the time domain. Both frequency and time domain analyses prove that our system is immune from reasonable unwanted perturbation and fabrication error and maintains stable operation. It is important to note that the stability issue inherently relies on the measurement circuit (where the generator, source, and load impedance are included), rather than the isolated PT-symmetric tunneling system.

### 4.3. Measurement Setup

The scattering properties of our device are measured with the Agilent Technologies network analyzer with part number E5071C, which can analyze *S* parameters from 9 kHz to 8.5 GHz with great precision. One of the DC bias ports of the amplifier is grounded, and the other port is supplied with a 1.3 V DC voltage. The source is from Agilent Technologies with part number E3631A. When the measurement range is from 20 MHz to 80 MHz, the step size of the excitation signal is 0.0375 MHz.

## Data Availability

All data needed to evaluate the conclusions in the paper are present in the paper. Additional data related to this paper may be requested from the authors.

## Supplementary Materials

Supplementary Materials 1. Scattering properties of the parity-time- (PT-) symmetric wave tunneling and information transfer system. 2. Stability analysis. Extended data Fig. S1: pole distributions of transfer function with parameter detuning. Extended data Fig. S2: numerical calculation of the impulse responses with parameter detuning. Extended data Fig. S3: comparison between experimental *S* parameters and simulation *S* parameters. Extended data Fig. S4: a closer look into the *S* parameter, ranging from 40 MHz to 50 MHz. Extended data Fig. S5: linearity of the tunneling device. Fig. S6: scattering parameters of the ideal PT-symmetric wave tunneling and information transfer device. Fig. S7: scattering parameters of the PT-symmetric wave tunneling and information transfer device by using the ADS and Modelithics package. Fig. S8: pole locations of transfer function with parameter detuning. Fig. S9: ADS simulation of impulse response with parameter detuning. Fig. S10: temporal responses at the source, obstacle, and load nodes with the static and dynamic obstacle.
