# Erratum to “Microwave Tunneling and Robust Information Transfer Based on Parity-Time-Symmetric Absorber-Emitter Pairs”

**DOI:** 10.34133/2020/3246023

**Published:** 2020-03-20

**Authors:** Zhicheng Xiao, Younes Ra'di, Sergei Tretyakov, Andrea Alù

**Affiliations:** ^1^Department of Electrical and Computer Engineering, The University of Texas at Austin, Austin, TX 78712, USA; ^2^Advanced Science Research Center, City University of New York, New York, NY 10031, USA; ^3^Department of Electronics and Nanoengineering, Aalto University, FI-00076 Aalto, Finland

In the article titled “Microwave Tunneling and Robust Information Transfer Based on Parity-Time-Symmetric Absorber-Emitter Pairs” [1], there were errors in Figure 2 which occurred during production. In panel (c), the red line should be attributed to “Re (ZNIC/Z0)” and the blue line should be attributed to “Im (ZNIC/Z0).” In panel (d), the blue line should read “∣S 21∣ = ∣S12∣” and the black line should read “|S22|.” The corrected figure is shown as Figure 1 below.

## Figures and Tables

**Figure 1:**
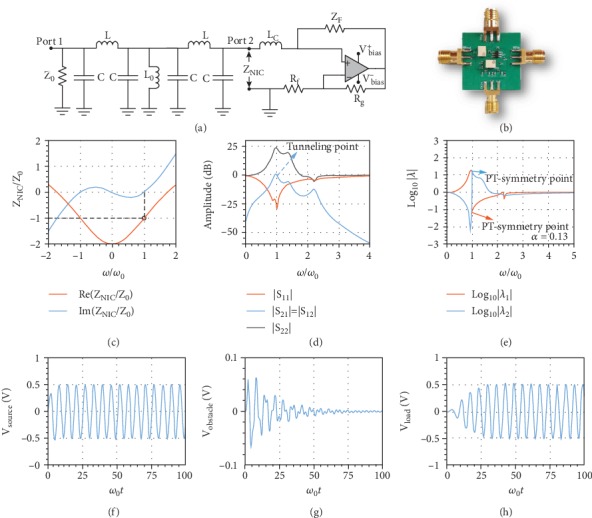
Realistic design and implementation of the PT-symmetric wave tunneling prototype. (a) Circuit schematic. Transmission line is replaced with a *π*-type transmission line which consists of inductor L and capacitor C. (b) Photograph of the fabricated PCB prototype. Two big white components are tunable resistors. The black component with six pins is the OPA 355-Q1 amplifier. Left and right ports are source port 1 and load port 2 in the schematic. Upper and lower ports are DC bias ports for the amplifier. (c) Dispersion of the impedance of the gain element. Black circle marks the operational point. (d) ADS and Modelithics simulation of the amplitude of scattering parameters. Tunneling point is marked in the figure. (e) Spectral properties of eigenvalues of the scattering matrix. Exact PT symmetry is achieved at tunneling frequency where eigenvalues obey unitary conditions |*λ*_1_(*ω*_0_)*λ*_2_(*ω*_0_)| = 1. The coupling coefficient *α* is 0.13, ensuring robust operation of the whole circuit in the presence of the obstacle. (f) Numerical transient response at the source port where full absorption is achieved at tunneling frequency. The generator voltage is 1 volt. (g) Numerical transient response at the obstacle which is short in the steady state. (h) Numerical transient response at the load port where full-wave tunneling is observed in the steady state.

## References

[1] Xiao Z., Ra’di Y., Tretyakov S., Alù A. (2019). Microwave tunneling and robust information transfer based on parity-time-symmetric absorber-emitter pairs. *Research*.

