# Supplementary material for: Microwave Tunneling and Robust Information Transfer Based on Parity-Time-Symmetric Absorber-Emitter Pairs
Source: Research (Wash D C). 2019 Nov 28;2019:7108494. doi: 10.34133/2019/7108494 (PMC6946254; doi:10.34133/2019/7108494)
Supplement: Supplementary Materials — 1. Scattering properties of the parity-time- (PT-) symmetric wave tunneling and information transfer system. 2. Stability analysis. Extended data Fig. S1: pole distributions of transfer function with parameter detuning. Extended data Fig. S2: numerical calculation of the impulse responses with parameter detuning. Extended data Fig. S3: comparison between experimental S parameters and simulation S parameters. Extended data Fig. S4: a closer look into the S parameter, ranging from 40 MHz to 50 MHz. Extended data Fig. S5: linearity of the tunneling device. Fig. S6: scattering parameters of the ideal PT-symmetric wave tunneling and information transfer device. Fig. S7: scattering parameters of the PT-symmetric wave tunneling and information transfer device by using the ADS and Modelithics package. Fig. S8: pole locations of transfer function with parameter detuning. Fig. S9: ADS simulation of impulse response with parameter detuning. Fig. S10: temporal responses at the source, obstacle, and load nodes with the static and dynamic obstacle. [file 7108494.f1.pdf]

Supplementary Materials
This file includes:
Supplementary text

Figures S1 to S10

Contents

Supplementary Materials 1

This file includes: 1

1. Scattering properties of parity-time (PT) symmetric wave tunneling and information transfer system 2

1.1 Ideal PT-symmetric wave tunneling and information transfer circuit 2

1.2 PT-symmetric wave tunneling and information transfer circuit with one-pole NIC 4

2. Stability analysis 4

2.1 Stability analysis of an ideal PT-symmetric wave tunneling and information transfer circuit 4

2.2 Robustness analysis with dynamic obstacle 6

Figures 7

1. Scattering properties of parity-time (PT) symmetric wave tunneling and information transfer system

1.1 Ideal PT-symmetric wave tunneling and information transfer circuit

The transfer matrix of the two-port network can be expressed as:

$$T=\begin{bmatrix}1 & 0\\ \frac{1}{Z_0} & 1\end{bmatrix}\begin{bmatrix}\cos\omega\tau & jZ_0\sin\omega\tau\\ \frac{j}{Z_0}\sin\omega\tau & \cos\omega\tau\end{bmatrix}\begin{bmatrix}1 & 0\\ \frac{1}{j\omega L_0} & 1\end{bmatrix}\begin{bmatrix}\cos\omega\tau & jZ_0\sin\omega\tau\\ \frac{j}{Z_0}\sin\omega\tau & \cos\omega\tau\end{bmatrix}\begin{bmatrix}1 & 0\\ -\frac{1}{Z_0} & 1\end{bmatrix}, \qquad (1)$$

where Z_0 is the characteristic impedance of the transmission line, τ is the time delay in the transmission line, ω is the signal frequency, L_0 is the inductance of the shunt inductor. By simplifying Eq. (1), the following four matrix elements are obtained:

$$\begin{cases}T_{11}=\cos 2\omega\tau+\frac{Z_0}{2L_0\omega}\sin 2\omega\tau+j\left[-\frac{Z_0}{2L_0\omega}+\frac{Z_0}{2L_0\omega}\cos 2\omega\tau-\sin 2\omega\tau\right],\\ T_{12}=jZ_0\left[\frac{Z_0}{2L_0\omega}+\sin 2\omega\tau-\frac{Z_0}{2L_0\omega}\cos 2\omega\tau\right],\\ T_{21}=\frac{-j}{L_0\omega},\\ T_{22}=\cos 2\omega\tau+\frac{Z_0}{2L_0\omega}\sin 2\omega\tau+j\left[\frac{Z_0}{2L_0\omega}-\frac{Z_0}{2L_0\omega}\cos 2\omega\tau+\sin 2\omega\tau\right].\end{cases} \qquad (2)$$

According to the relation between scattering matrix and transfer matrix:

$$S=\frac{1}{T_{11}+T_{12}/Z_0+T_{21}Z_0+T_{22}}\begin{bmatrix}T_{11}+T_{12}/Z_0-T_{21}Z_0-T_{22} & 2\left(T_{11}T_{22}-T_{12}T_{21}\right)\\ 2 & -T_{11}+T_{12}/Z_0-T_{21}Z_0+T_{22}\end{bmatrix}=\begin{bmatrix}S_{11} & S_{12}\\ S_{21} & S_{22}\end{bmatrix}, \qquad (3)$$

S-parameters are given by

$$\begin{cases}S_{11}=\frac{2j\cos\omega\tau\left(-2\omega L_0\sin\omega\tau+Z_0\cos\omega\tau\right)}{-jZ_0+\left(4\omega L_0-jZ_0\right)\cos 2\omega\tau+2\left(j\omega L_0+Z_0\right)\sin 2\omega\tau},\\ S_{12}=S_{21}=\frac{4\omega L_0}{-jZ_0+\left(4\omega L_0-jZ_0\right)\cos 2\omega\tau+2\left(j\omega L_0+Z_0\right)\sin 2\omega\tau},\\ S_{22}=\frac{6L_0\omega\sin 2\omega\tau+Z_0\left(2+6\sin^2\omega\tau\right)}{2L_0\omega\left(-2j\cos 2\omega\tau+\sin 2\omega\tau\right)-Z_0\left(1+\cos 2\omega\tau+2j\sin 2\omega\tau\right)}.\end{cases} \qquad (4)$$

When $\tau=\frac{1}{4f_0}=\frac{\pi}{2\omega_0}$, the above equations can be simplified to

$$\begin{cases} S_{11} = \dfrac{-\left(1+\cos\dfrac{\pi\omega}{\omega_0}\right)+\dfrac{2\alpha\omega}{\omega_0}\sin\dfrac{\pi\omega}{\omega_0}}{1+\left(1+j\dfrac{4\alpha\omega}{\omega_0}\right)\cos\dfrac{\pi\omega}{\omega_0}+2\left(j-\dfrac{\alpha\omega}{\omega_0}\right)\sin\dfrac{\pi\omega}{\omega_0}}, \\ S_{12} = S_{21} = \dfrac{j\dfrac{4\alpha\omega}{\omega_0}}{1+\left(1+j\dfrac{4\alpha\omega}{\omega_0}\right)\cos\dfrac{\pi\omega}{\omega_0}+2\left(j-\dfrac{\alpha\omega}{\omega_0}\right)\sin\dfrac{\pi\omega}{\omega_0}}, \\ S_{22} = \dfrac{-\dfrac{6\alpha\omega}{\omega_0}\sin\dfrac{\pi\omega}{\omega_0}+3\cos\dfrac{\pi\omega}{\omega_0}-5}{1+\left(1+j\dfrac{4\alpha\omega}{\omega_0}\right)\cos\dfrac{\pi\omega}{\omega_0}+2\left(j-\dfrac{\alpha\omega}{\omega_0}\right)\sin\dfrac{\pi\omega}{\omega_0}}, \end{cases} \tag{5}$$

where ω_0 is the designed frequency of the circuit, $\alpha = \dfrac{\omega_0 L_0}{Z_0}$ is the ratio between the impedance of the inductor (obstacle) and the characteristic impedance. In a two-port PT-symmetric system, to have a unitary transmission, at least one of the reflection coefficients must be zero. When $S_{11} = 0$, there are two sets of solutions

$$\begin{cases} \dfrac{\pi\omega}{\omega_0} = (2k+1)\pi, k = 0,\ 1,\ 2,\ 3\cdots \\ \dfrac{1}{2\alpha}\left(\dfrac{\omega}{\omega_0}\right)^{-1} = \tan\left(\dfrac{\pi}{2}\dfrac{\omega}{\omega_0}\right). \end{cases} \tag{6}$$

In the case $\dfrac{\omega}{\omega_0} = 2k+1,\ k = 0,\ 1,\ 2,\ 3$, the scattering matrix downgrades to

$$S = \begin{bmatrix} 0 & -1 \\ -1 & -\dfrac{2j}{\alpha}\left(\dfrac{\omega}{\omega_0}\right)^{-1} \end{bmatrix}. \tag{7}$$

In the case $\dfrac{1}{2\alpha}\left(\dfrac{\omega}{\omega_0}\right)^{-1} = \tan\left(\dfrac{\pi}{2}\dfrac{\omega}{\omega_0}\right)$, the scattering matrix downgrades to

$$S = \begin{bmatrix} 0 & 1 \\ 1 & \dfrac{2j}{\alpha}\left(\dfrac{\omega}{\omega_0}\right)^{-1} \end{bmatrix}. \tag{8}$$

In summary, the solution $\dfrac{\omega}{\omega_0} = 2k+1,\ k = 0,\ 1,\ 2,\ 3$ corresponds to the odd scattering state, while the solution $\dfrac{1}{2\alpha}\left(\dfrac{\omega}{\omega_0}\right)^{-1} = \tan\left(\dfrac{\pi}{2}\dfrac{\omega}{\omega_0}\right)$ corresponds to the even scattering state. The transmission phase of Eqs. (7) and (8) confirms this assessment. It is worth mentioning that the PT tunneling resonance is irrelevant to whether if the system is in exact PT phase or in broken PT phase regimes. When the loss and gain is balanced, the wave tunneling in PT-symmetric system is very similar to electron or photon tunneling phenomena.

Meanwhile, the wave tunneling can also happen on the second port where $S_{22}=0$. The solutions are

$$\left(\frac{\omega}{\omega_0}\right)^{-1}=\frac{6\alpha\sin\pi\dfrac{\omega}{\omega_0}}{3\cos\pi\dfrac{\omega}{\omega_0}-5}. \tag{9}$$

As we see from the above equation, the solutions always exist. But the tunneling frequencies are inherently different from Eq. (6). The smaller α is, the larger ω becomes. In this case, the $|S_{12}|=|S_{21}|=1$ and $S_{11}\neq 0$.

Figure. S6 demonstrates the scattering parameters of an ideal PT-symmetric wave tunneling and information transfer device. There are ten forward unidirectional tunneling points. They are: $\frac{\omega}{\omega_0}=1,\ 3,\ 5,\ 7,\ 9$ and $\frac{\omega}{\omega_0}=0.88,\ 2.68,\ 4.53,\ 6.42,\ 8.35$. There are four backward wave tunneling points, they are: $\frac{\omega}{\omega_0}=7.56,\ 7.82,\ 9.44,\ 9.88$. These tunneling points are consistent with Eqs. (6) and (9).

1.2 PT-symmetric wave tunneling and information transfer circuit with one-pole NIC

For practical implementation of the PT-symmetric wave tunneling and information transfer, we make two major modifications over the ideal model. First, the transmission line is replaced with a π model LC tank with finite transmission window to shrink the form factor of the board. Second, the negative impedance is implemented with an amplifier feedback circuit.

We apply the transfer matrix formalism:

$$T=\begin{bmatrix}1 & 0\\ \dfrac{1}{Z_0} & 1\end{bmatrix}\begin{bmatrix}1 & 0\\ j\omega C & 1\end{bmatrix}\begin{bmatrix}1 & j\omega L\\ 0 & 1\end{bmatrix}\begin{bmatrix}1 & 0\\ j\omega C & 1\end{bmatrix}\begin{bmatrix}1 & 0\\ \dfrac{1}{j\omega L_0} & 1\end{bmatrix}\begin{bmatrix}1 & 0\\ j\omega C & 1\end{bmatrix}\begin{bmatrix}1 & j\omega L\\ 0 & 1\end{bmatrix}\begin{bmatrix}1 & 0\\ j\omega C & 1\end{bmatrix}\begin{bmatrix}1 & 0\\ \dfrac{1}{Z_{NIC}} & 1\end{bmatrix}. \tag{10}$$

The corresponding scattering matrix can be obtained by substitute the above equation into Eq. (3). Implementation of the PCB board involves consideration of parasitic effects, wave leakage in the circuit channel and many more. In this part, we demonstrate our simulation results of our PCB board with ADS and Modelithics package. Figure. S7 demonstrates our PCB board simulation results. The tunneling frequency is 48.7 MHz, where the transmission $|S_{12}|=|S_{21}|=0.01\text{ dB}$, and reflection $|S_{11}|=-28.8\text{ dB}$, $|S_{22}|=23.4\text{ dB}$. The simulation results are in excellent agreement with our theoretical prediction in previous section.

2. Stability analysis

2.1 Stability analysis of an ideal PT-symmetric wave tunneling and information transfer circuit

In this section, we provide a detailed derivation of the transfer function and stability analysis of an ideal PT-symmetric wave tunneling circuit. To analyze the influence of parameter detuning, we assume two small perturbations on the circuit: relative time delay perturbation $\frac{\Delta\tau}{\tau}$ on the second segment of transmission and small perturbation $\frac{\Delta Z}{Z_0}$ on the ideal negative impedance.

At port 2, the effective load impedance is a negative impedance in parallel with the characteristic impedance $\left[1+\left(\frac{\Delta Z}{Z_0}\right)^{-1}\right] Z_0$. Therefore, the effective reflection coefficient on the load side is

$$\Gamma_{l_2} = \frac{1}{1+2\frac{\Delta Z}{Z_0}}. \tag{11}$$

The input impedance on the second transmission line is

$$Z_{in2} = Z_0 \frac{e^{\tau_2 s} + \Gamma_{l_2} e^{-\tau_2 s}}{e^{\tau_2 s} - \Gamma_{l_2} e^{-\tau_2 s}}, \tag{12}$$

where τ_2 is the time delay of the second transmission line. For the first transmission line, the load impedance is an inductor in parallel with input impedance on the second transmission line

$$Z_{L_1} = sL_0 \,\square\, \text{[illegible]} \tag{13}$$

where $\square$ is an operator to calculate the shunt impedance on the left and right side of this symbol. Then, the load impedance on the first transmission line can be simplified to

$$Z_{L_1} = \frac{sZ_0 L_0 \left(e^{\tau_2 s} + \Gamma_{l_2} e^{-\tau_2 s}\right)}{\left(Z_0 + sL_0\right) e^{\tau_2 s} + \left(Z_0 - sL_0\right) \Gamma_{l_2} e^{-\tau_2 s}}. \tag{14}$$

So, for the first transmission line the reflection coefficient is

$$\Gamma_{l_1} = \frac{-Z_0 e^{\tau_2 s} + \left(2sL_0 - Z_0\right) \Gamma_{l_2} e^{-\tau_2 s}}{\left(2sL_0 + Z_0\right) e^{\tau_2 s} + Z_0 \Gamma_{l_2} e^{-\tau_2 s}}. \tag{15}$$

The transfer function on the inductor can be expressed as

$$H_{inductor}\left(s\right) = \frac{1+\Gamma_{l_1}}{3e^{\tau_1 s} + \Gamma_{l_1} e^{-\tau_1 s}}, \tag{16}$$

where τ_1 is the time delay on the first transmission line. As a result, the transfer function which is defined as the voltage ratio on the negative impedance versus the generator can be written as

$$H\left(s\right) = \frac{1+\Gamma_{l_1}}{3e^{\tau_1 s} + \Gamma_{l_1} e^{-\tau_1 s}} \times \frac{1+\Gamma_{l_2}}{e^{\tau_2 s} + \Gamma_{l_2} e^{-\tau_2 s}}, \tag{17}$$

where Γ_{l_1} is expressed in Eq. (15). We continue to simplify the transfer function to

$$H\left(s\right) = \frac{4\left(1+\frac{\Delta Z}{Z_0}\right) L_0 s}{3\left(1+2\frac{\Delta Z}{Z_0}\right)\left(2L_0 s + Z_0\right) e^{(\tau_1+\tau_2)s} + 3Z_0 e^{(\tau_1-\tau_2)s} - Z_0\left(1+2\frac{\Delta Z}{Z_0}\right) e^{(\tau_2-\tau_1)s} + \left(2L_0 s - Z_0\right) e^{-(\tau_1+\tau_2)s}}. \tag{18}$$

Now, we assume $\tau_1 = \tau$ and $\tau_2 = \tau\left(1+\frac{\Delta\tau}{\tau}\right)$. The above equation will be simplified to

$$H(s)=\frac{4\left(1+\frac{\Delta Z}{Z_0}\right)L_0 s}{3\left(1+2\frac{\Delta Z}{Z_0}\right)(2L_0 s+Z_0)e^{\tau\left(2+\frac{\Delta\tau}{\tau}\right)s}+3Z_0e^{-\Delta\tau s}-Z_0\left(1+2\frac{\Delta Z}{Z_0}\right)e^{\Delta\tau s}+(2L_0 s-Z_0)e^{-\tau\left(2+\frac{\Delta\tau}{\tau}\right)s}}. \tag{19}$$

The above equation is the transfer function of an ideal PT-symmetric wave tunneling and information transfer circuit with parameter detuning. We apply simple numerical calculations and figure out the poles of the transfer function.

Figure S8 demonstrates the pole locations with parameter detuning. Our study indicates that the circuit remains stable for any value $\alpha > 0$ based on the assumption that there is no parameter detuning. If imperfection exists, the coupling coefficient α should be larger than 0.1 to ensure stable operation.

It is also important to verify the stability issue in time domain. Figure S9 demonstrates the impulse responses of the scattering systems with various detuning schemes. The finite amplitude of impulse responses is consistent with the numerical results in Fig. S8.

In summary, the ideal PT-symmetric tunneling circuit is stable for any obstacle strength. To ensure a more robust operation [5% tolerance on impedance and delay detuning], the coupling coefficient should be larger than 0.1. It is important to note that the EP is irrelevant to the stability issue.

2.2 Robustness analysis with dynamic obstacle

In this section, we will graphically prove the robustness of system with dynamic obstacle. From Figs S10a-c, we can see that the system will reach steady state in 0.5 μs when the obstacle is static. In Figs S10d-f, we apply a square wave modulation on the obstacle at 0.5 μs. The source and load sides will reach to steady in a longer time, which is approximately 1 μs. These graphs prove that our system is capable of retuning to steady state when the obstacle dynamically changes in time.

Figures

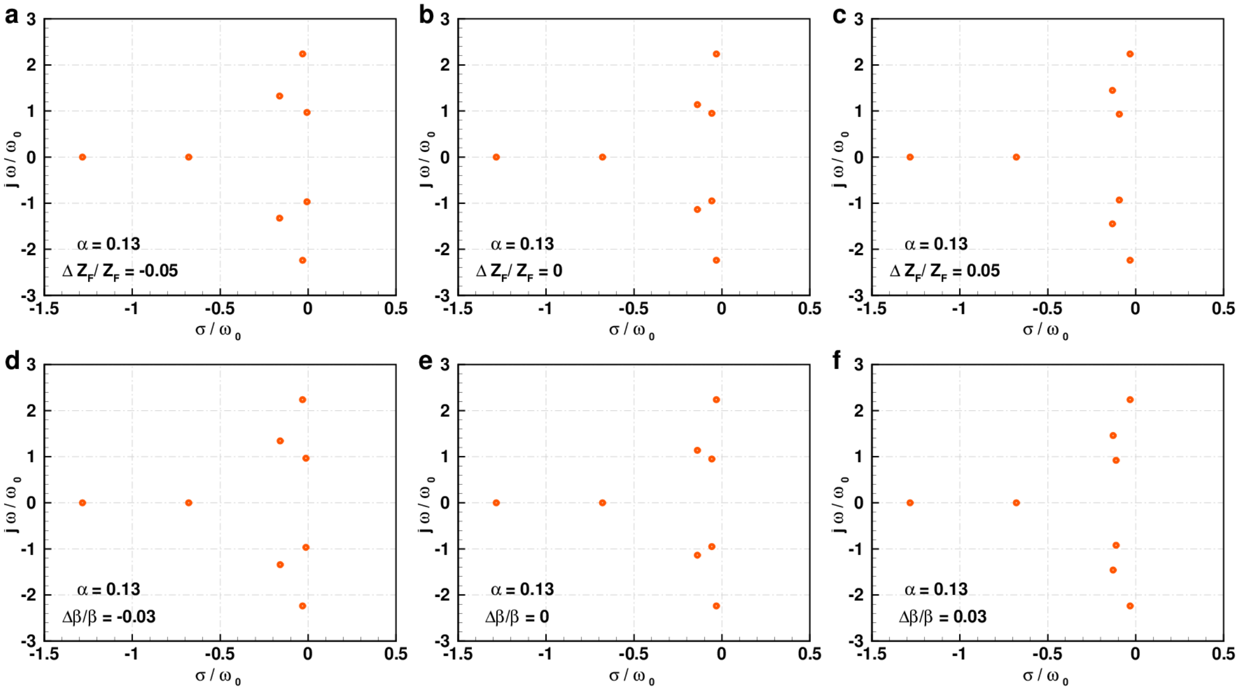

Extended Data Fig. S1. Pole distributions of transfer function with parameter detuning. **a** Pole distribution with $\alpha = 0.13,\ \frac{\Delta Z_F}{Z_F} = -0.05$. **b** Pole distribution with $\alpha = 0.13,\ \frac{\Delta Z_F}{Z_F} = 0$. **c** Pole distribution with $\alpha = 0.13,\ \frac{\Delta Z_F}{Z_F} = 0.05$. **d** Pole distribution with $\alpha = 0.13,\ \frac{\Delta \beta}{\beta} = -0.03$. **e** Pole locations with $\alpha = 0.13,\ \frac{\Delta \beta}{\beta} = 0$. **f** Pole distribution with $\alpha = 0.13,\ \frac{\Delta \beta}{\beta} = 0.03$. There are 8 poles due to the finite bandwidth of the π -model transmission line.

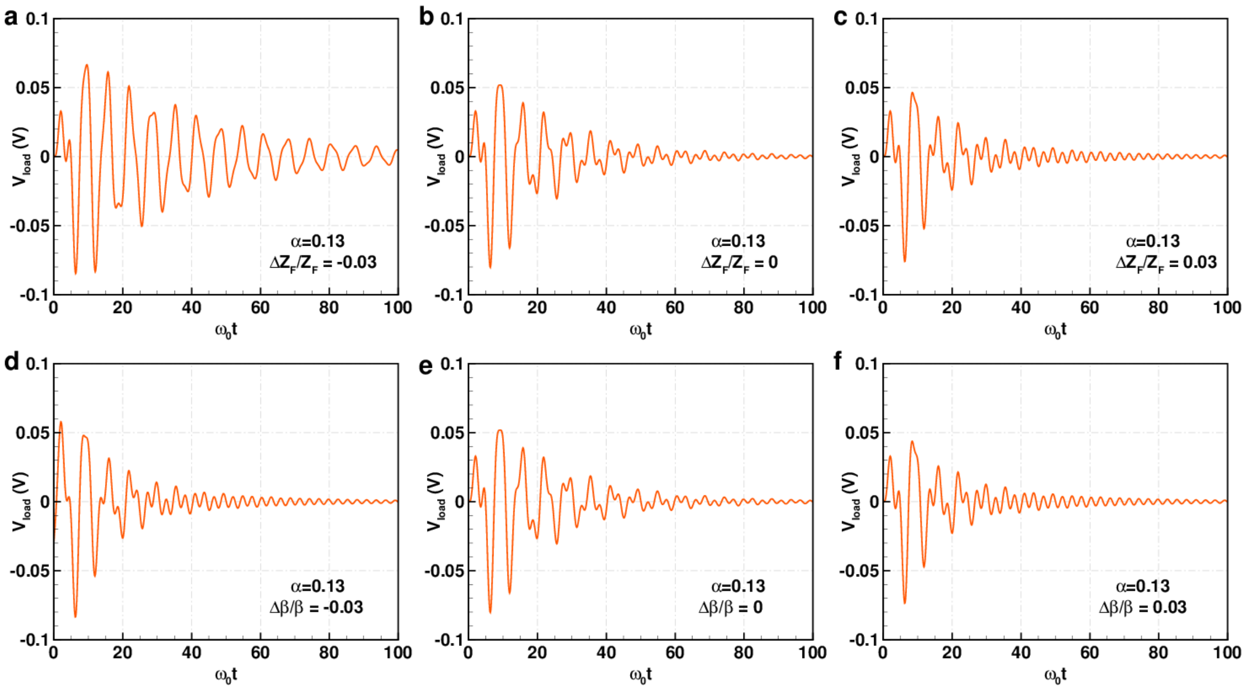

Extended Data Fig. S2. Numerical calculation of the impulse responses with parameter detuning. The input pulse has an amplitude of 1 V. **a** Impulse response with $\alpha = 0.13,\ \frac{\Delta Z_F}{Z_F} = -0.03$. **b** Impulse response with

$\alpha = 0.13, \ \frac{\Delta Z_F}{Z_F} = 0$. **c** Impulse response with $\alpha = 0.13, \ \frac{\Delta Z_F}{Z_F} = 0.03$. **d** Impulse response with $\alpha = 0.13, \ \frac{\Delta \beta}{\beta} = -0.03$. **e** Impulse response with $\alpha = 0.13, \ \frac{\Delta \beta}{\beta} = 0$. **f** Impulse response with $\alpha = 0.13, \ \frac{\Delta \beta}{\beta} = 0.03$.

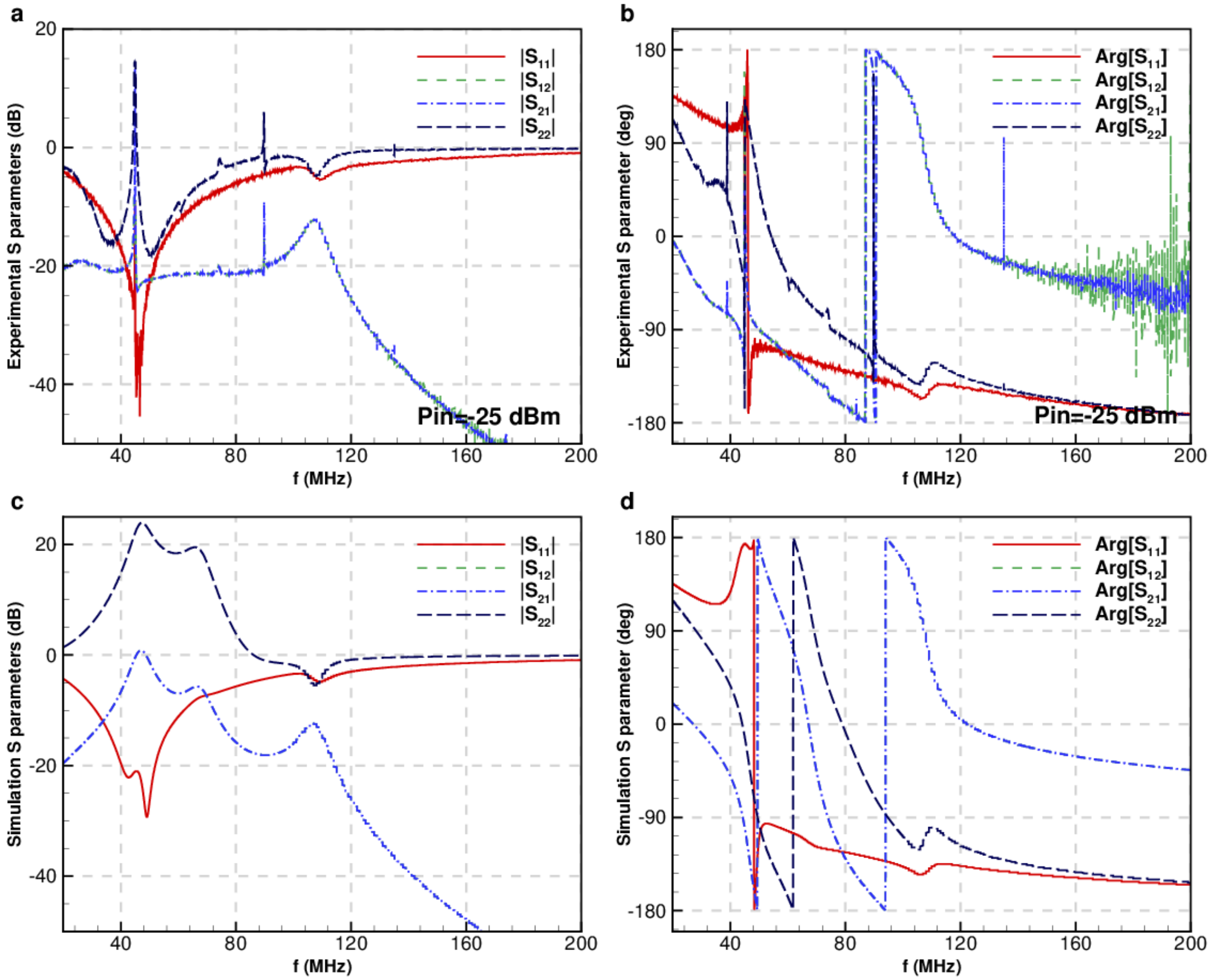

Extended Data Fig. S3. Comparison between experimental S parameters and simulation S parameters. The input wave power is -25 dBm. **a** Amplitude of experimental scattering parameters ranging from 20 MHz to 200 MHz. **b** Phase of experimental scattering parameters ranging from 20 MHz to 200 MHz. **c** Amplitude of simulation scattering parameters ranging from 20 MHz to 200 MHz. **d** Phase of simulation scattering parameters ranging from 20 MHz to 200 MHz.

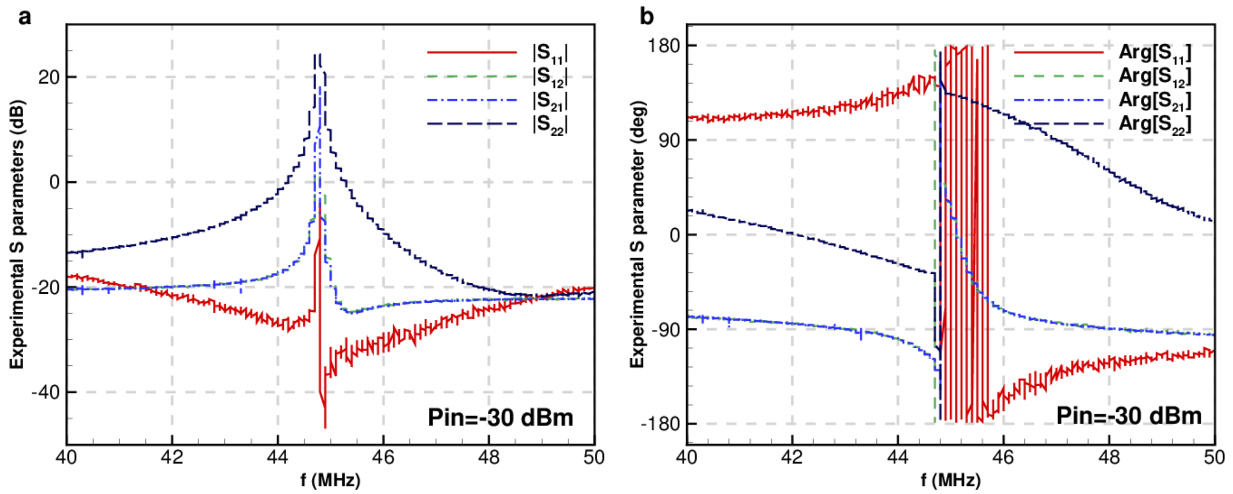

Extended Data Fig. S4. A closer look into the S parameter, ranging from 40 MHz to 50 MHz. **a** Amplitude of experimental scattering parameters. **b** Phase of experimental scattering parameters. Phase of reflection coefficient S_{11} experiences large oscillations from 44 to 46 MHz. This is due to the undefined phase at singular point.

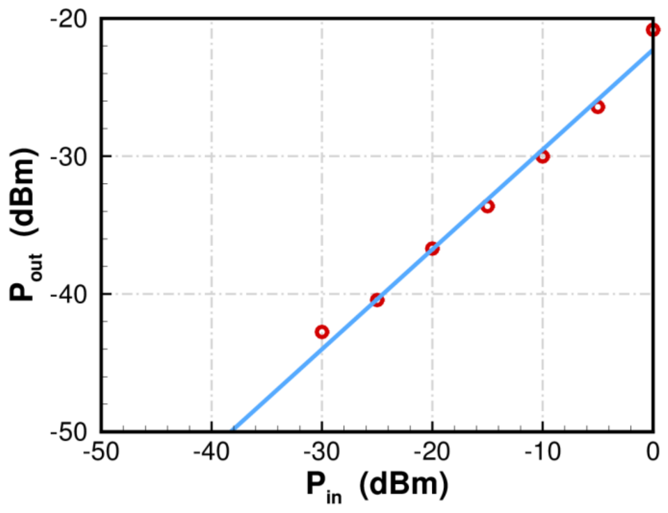

Extended Data Fig. S5. Linearity of the tunneling device. Straight line represents the fitting curve of the input-output power relation. Dots are measured experimental data. The system shows an excellent linear power response from -25 dBm to -5 dBm.

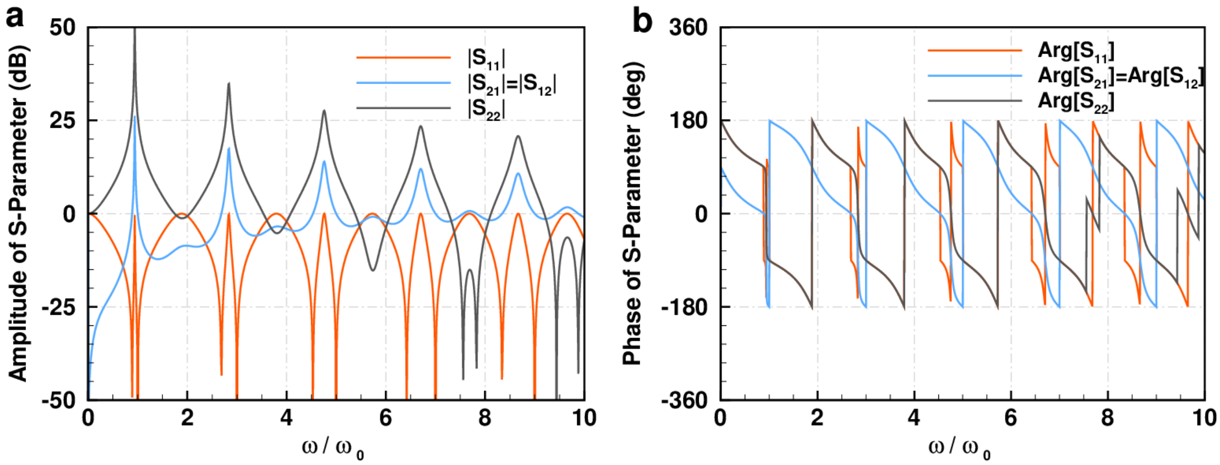

Fig. S6 Scattering parameters of ideal PT-symmetric wave tunneling and information transfer device. We choose $\alpha = 0.1$ in the above graph. **a**. The amplitude of scattering parameters. **b**. The phase of scattering parameters.

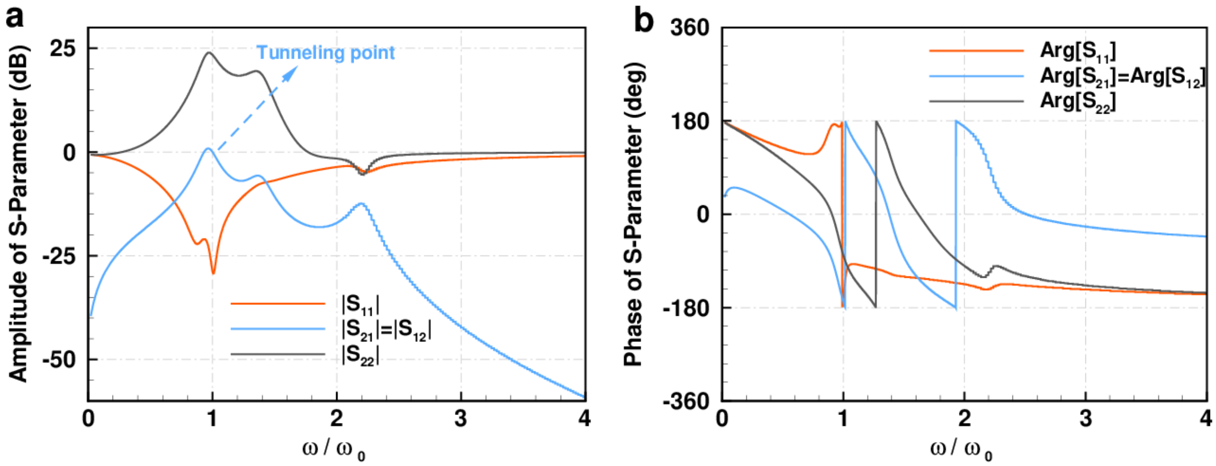

Fig. S7 Scattering parameters of PT-symmetric wave tunneling and information transfer device by using ADS and Modelithics package. **a**. The amplitude of scattering parameters. **b**. The phase of scattering parameters. The following components are used: $L = 150\text{nH}$, $C = 68\text{pF}$, $L_0 = 20nH$, $L_C = 240nH$, and the amplifier is Texas Instrument (TI) OPA355. The resistors on the converting input port of the amplifier are $R = 560\ \Omega$, which are tunable. The theoretical operational frequency is $f_0 = \frac{\omega_0}{2\pi} = \frac{1}{2\pi\sqrt{LC}} = 49.8\ \text{MHz}$. The tunneling frequency inferred from the simulation results is 48.7 MHz, which is very close to the theoretical value. The parameter $\alpha = \frac{\omega_0 L_0}{Z_0}$ is 0.13 in our circuit.

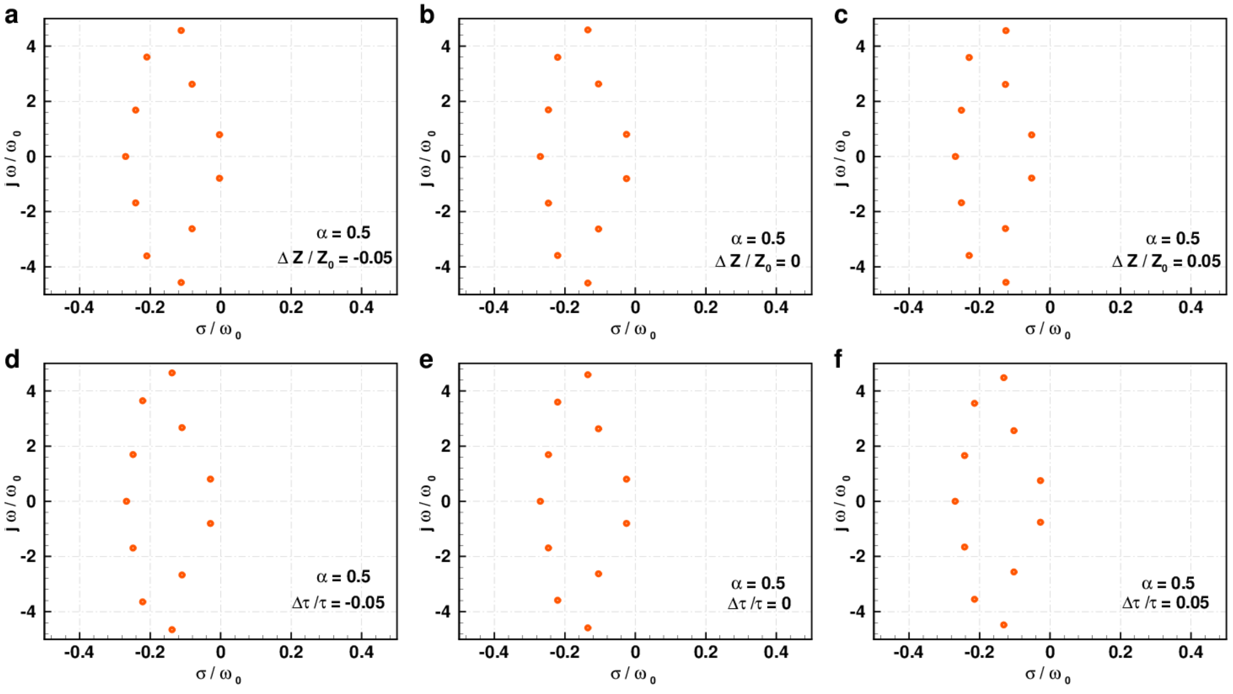

Fig. S8. Pole locations of transfer function with parameter detuning. **a**. Pole locations with $\alpha = 0.5,\ \frac{\Delta Z}{Z_0} = -0.05$. **b**. Pole locations with $\alpha = 0.5,\ \frac{\Delta Z}{Z_0} = 0$. **c**. Pole locations with $\alpha = 0.5,\ \frac{\Delta Z}{Z_0} = 0.05$. **a**. Pole locations with $\alpha = 0.5,\ \frac{\Delta \tau}{\tau} = -0.05$. **b**. Pole locations with $\alpha = 0.5,\ \frac{\Delta \tau}{\tau} = 0$. **c**. Pole locations with $\alpha = 0.5,\ \frac{\Delta \tau}{\tau} = 0.05$. Note that the number of poles is infinite, and we only show the poles that will potentially cause stability problems.

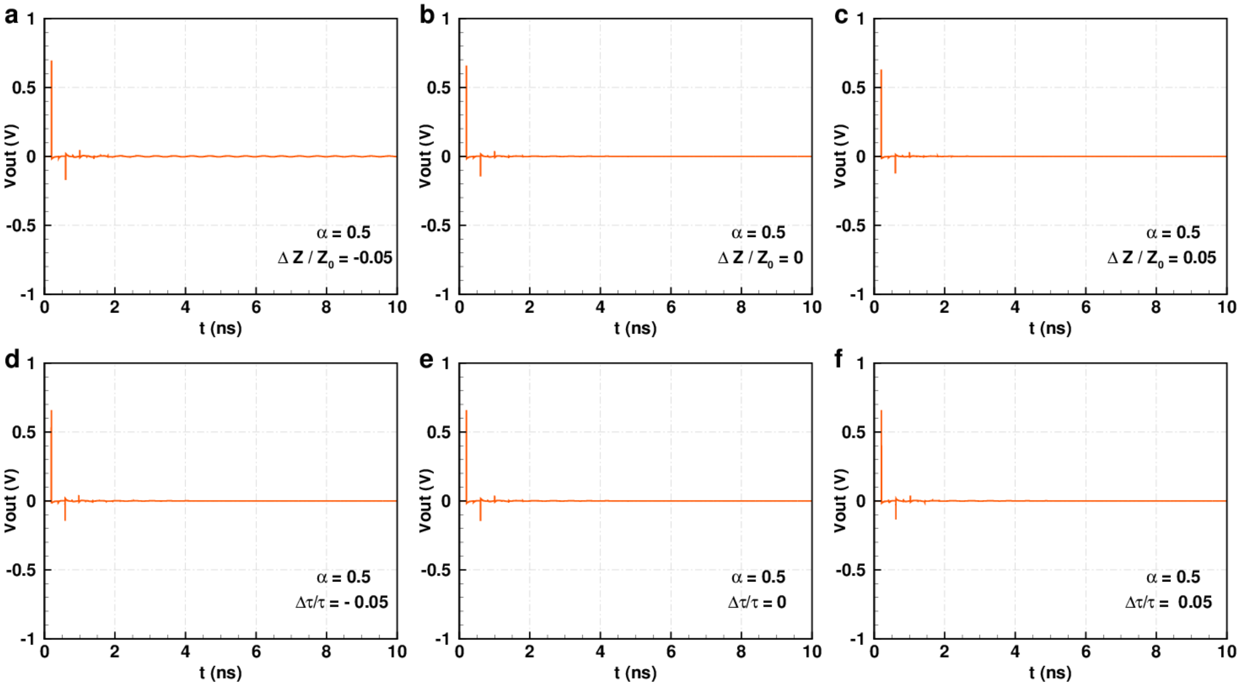

Fig. S9. ADS simulation of impulse response with parameter detuning. The input pulse has an amplitude of 1 V and 1 *ps* duration. **a**. Impulse response with $\alpha = 0.5,\ \frac{\Delta Z}{Z_0} = -0.05$. **b**. Impulse response with $\alpha = 0.5,\ \frac{\Delta Z}{Z_0} = 0$.

c. Impulse response with $\alpha = 0.5$, $\frac{\Delta Z}{Z_0} = 0.05$. **a**. Impulse response with $\alpha = 0.5$, $\frac{\Delta \tau}{\tau} = -0.05$. **b**. Impulse response with $\alpha = 0.5$, $\frac{\Delta \tau}{\tau} = 0$. **c**. Impulse response with $\alpha = 0.5$, $\frac{\Delta \tau}{\tau} = 0.05$.

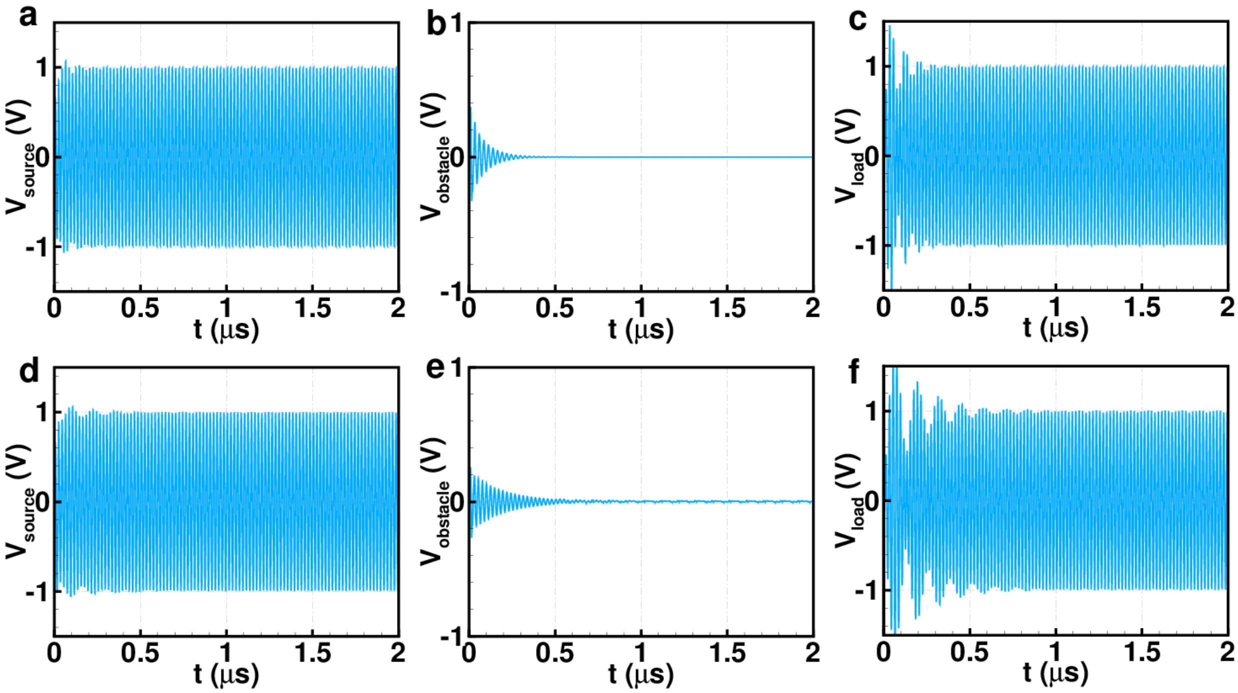

Fig. S10. Temporal responses at source, obstacle, and load nodes with static and dynamic obstacle. We assume that the time delay on the quarter-wavelength transmission line is . $\tau = 5$ ns ., the static obstacle inductor is $L_0 = 100$ nH , and the characteristic impedance is $Z_0 = 50\ \Omega$. The dynamic obstacle is $L_0(t) = \frac{L_0}{2}\left[1 + \sum_{n=0}^{\infty} \mathrm{rect}(t - t_0 - nT_m)\right]$, where $t_0 = 0.5$ μs, $T_m = 0.1$ μs . **a**, Numerical transient response at the source port where full absorption is achieved at tunneling frequency with static obstacle. **b**, Numerical transient response at the obstacle which is short in the steady state with static obstacle. **c**, Numerical transient response at the load port where full wave tunneling is observed in the steady state with static obstacle. **d**, Numerical transient response at the source port with dynamic obstacle. **e**, Numerical transient response at the obstacle with dynamic obstacle. **f**, Numerical transient response at the load port where full wave tunneling is observed in the steady state with dynamic obstacle.
